# A Practical Framework for Wastewater-Based Monitoring of Substance Use in Public Health Settings

**DOI:** 10.3390/ijerph23040518

**Published:** 2026-04-17

**Authors:** Shisbeth Tabora-Sarmiento, Thomas D. Sinkway, Sarah E. Robinson, Francisco Paneque, Nicole Winn, Jeantel Cheramy, Linda B. Cottler, John A. Bowden, Tara Sabo-Attwood, Joseph H. Bisesi

**Affiliations:** 1 Center for Aquatic and Invasive Plants, Institute of Food and Agricultural Sciences, University of Florida, Gainesville, FL 32653, USA; staborasarmiento@ufl.edu (S.T.-S.);; 2 Center for Environmental and Human Toxicology, College of Veterinary Medicine, University of Florida, Gainesville, FL 32611, USA; sinkwayt@ufl.edu (T.D.S.); sarah.robinson@ufl.edu (S.E.R.); jcheramy@ufl.edu (J.C.); john.bowden@ufl.edu (J.A.B.); 3 Department of Environmental and Global Health, College of Public Health and Health Professions, University of Florida, Gainesville, FL 32610, USA; 4 Department of Chemistry, College of Liberal Arts and Sciences, University of Florida, Gainesville, FL 32611, USA; 5 Department of Physiological Sciences, College of Veterinary Medicine, University of Florida, Gainesville, FL 32610, USA; 6 Department of Epidemiology, College of Public Health, Health Professions and Medicine, University of Florida, Gainesville, FL 32610, USA; lbcottler@ufl.edu; 7 Department of Environmental Health Sciences, Arnold School of Public Health, University of South Carolina, Columbia, SC 29208, USA; saboattw@mailbox.sc.edu; 8 Institute of Food and Agricultural Sciences, School of Forest, Fisheries and Geomatics Sciences, University of Florida, Gainesville, FL 32611, USA

**Keywords:** drugs, LC-MS/MS, wastewater

## Abstract

**Highlights:**

**Public health relevance—How does this work relate to a public health issue?**

- This study provides a complementary approach to overcome biases in traditional drug surveillance by using wastewater as an objective population-level data source.
- The practical framework enables rapid detection of emerging drug trends across communities.

**Public health significance—Why is this work of significance to public health?**

- This study establishes a scalable analytical method for simultaneous detection of dozens of drugs in wastewater.
- Our proposed workflow enhances reliability of wastewater surveillance by minimizing analyte loss and matrix interference.

**Public health implications—What are the key implications or messages for practitioners, policy makers and/or researchers in public health?**

- The workflow enables earlier detection of emerging substance use threats to guide rapid public health responses.
- It provides a transferable framework to strengthen national drug monitoring and policy decision-making.

**Abstract:**

The ongoing substance use crisis in the United States involves a broad range of illicit and prescription drugs, including opioids, stimulants, sedatives, and various psychoactive and non-psychoactive compounds. Traditional surveillance methods rely on self-reported data, which could lead to bias and recall inconsistencies. Wastewater-based epidemiology has emerged as a powerful, non-invasive tool for monitoring community-level drug use, offering near real-time estimates and the potential to serve as an early warning system. However, challenges such as analyte degradation, wastewater variability, and matrix effects can affect data quality and comparability across regions. This study presents a standardized, practical workflow for multi-drug (*n* = 52) detection in wastewater, aiming to minimize analyte loss and improve reproducibility. Composite samples were collected from multiple U.S. cities, transported on ice, and extracted using solid-phase extraction. Extraction efficiencies were compared using Oasis Hydrophilic-Lipophilic-Balanced and Mixed-mode Cation-Exchange (MCX) cartridges, with the MCX sorbent providing complementary reversed-phase and cation-exchange interactions that enabled the retention of chemically diverse compounds across multiple drug classes. Analysis was performed with an Ultra-High-Performance Liquid Chromatography system coupled to a triple quadrupole mass spectrometer, in which the instrument parameters and critical methodological considerations, including sample handling, transport, column selection, and method validation, are detailed. This work contributes to the development of a robust, scalable protocol for multi-drug surveillance in wastewater, supporting timely, data-driven public health responses and informing national drug policy efforts.

## 1. Introduction

Opioid use dates back to the 1800s, when these substances were introduced as an effective treatment for pain [1,2]. They quickly gained popularity among both physicians and patients. However, by the late 1880s, concerns began to emerge about the misuse and harmful effects of opioids, prompting tighter regulations and more restricted use [2,3], which led to a decline in opioid consumption in the early 1900s [2]. Despite this, a modern opioid epidemic remains closely tied to misuse. Although prescription rates have generally declined, there has been a troubling rise in the occurrence of fatal overdoses, initially involving heroin and, more recently, illicitly manufactured fentanyl and its highly potent analogues [1,2,4,5]. This opioid epidemic has impacted all aspects of society and the Center for Disease Control and Prevention estimates $78.5 billion a year as its economic burden related to healthcare, lost productivity, addiction treatment, and criminal justice [4,6].

Opioids are substances that interact with opioid receptors in the central nervous system. Opioids exist in various forms and can be classified as endogenous, naturally occurring, semi-synthetic, or fully synthetic. Natural opioids, such as morphine and codeine, are derived directly from the opium poppy, with morphine being the most widely used in clinical settings due to its potent analgesic properties. Semi-synthetic opioids, like heroin and oxycodone, are chemically modified versions of natural alkaloids. In contrast, fully synthetic opioids—such as methadone and fentanyl—have entirely different chemical structures from naturally occurring opioids but still bind to the same receptors, producing similar effects [1,2].

The substance use epidemic in the United States extends far beyond opioids. Other widely used substances include stimulants such as methamphetamine and cocaine, as well as other legal substances such as nicotine (used by >21% of Americans aged > 12 years old), alcohol (used by >50% of Americans aged > 12 years old), and cannabis (used by >17% of Americans aged > 12 years old)—the three most commonly used drugs in the country [4]. Nicotine use has evolved over time, with current trends showing that vaping nicotine and cannabis has become the most prevalent form of substance use among adolescents [7]. While cannabis (cannabidiol, CBD) is legally available for medical purposes, recreational marijuana remains the most commonly used illegal drug and is linked to increased risk of motor vehicle accidents [8]. Meanwhile, the use of stimulants, such as cocaine and amphetamines, continues to rise, with global cocaine manufacture rising by 56% between 2013 and 2016, and global seizures of amphetamine-related stimulants increasing by 20% between 2015 and 2016 [9]. The concurrent use of alcohol with other substances further compounds these dangers, significantly increasing the risk of overdose or the occurrence of accidental injury or death. These concerning trends highlight the urgent need for comprehensive surveillance systems and sustained monitoring to inform effective public health strategies.

Traditional surveillance on substance abuse typically involves self-reported data or the analysis of human biological samples. However, self-reported data can be subject to bias due to underreporting, social desirability, and recall inconsistencies. Additionally, the analysis of biological samples requires detailed care and adds privacy risks and concerns. Modern approaches, such as utilizing mass spectrometry, have been developed along with the use of different types of samples [10]. These newer techniques can aid in the earlier identification of substance abuse and emerging drugs. Moreover, the utilization of mass spectrometry on wastewater-based samples allows for the surveillance and ability to monitor drug abuse on a short time scale with precise measurements [10,11].

The use of wastewater has played a role in public health since the early 2000s, when the term wastewater-based epidemiology (WBE) was first used by the World Health Organization (WHO) in efforts to monitor the polio virus and confirm eradication in certain areas [12,13]. Since then, WBE has evolved into a valuable tool for tracking infectious disease and patterns of substance use. This emerging method is based on the analysis of chemicals and/or biomarkers in untreated wastewater from a given catchment area to obtain both qualitative and quantitative data on the activity of local populations. WBE offers several advantages, such as near-real time consumption estimates, and represents a more cost-effective approach to population testing that could become an early warning system [12,14,15].

The analysis of wastewater for illicit drug detection typically involves complex analytical procedures to acquire easily understandable results (e.g., identification and quantitation). Wastewater samples typically undergo an extraction, such as solid-phase extraction (SPE), to concentrate and purify drug analytes, followed by analysis using liquid-chromatography tandem mass spectrometry (LC-MS/MS) or gas chromatography tandem mass spectrometry (GC-MS/MS), though LC-MS/MS is more frequently used due to its robustness, high sensitivity, and high selectivity for most illicit drugs [16].

While WBE holds great promise for monitoring illicit drug use, its application presents several challenges. Analyte degradation during storage, variability in wastewater composition, and matrix effects during analysis can all compromise data quality and subsequent interpretation [17]. Current WBE studies often focus on single or limited drug classes and may not capture emerging substances or novel psychoactive compounds, which restricts the comprehensiveness of community-level drug surveillance. In addition, substantial variability in analytical methodologies, such as extraction techniques (namely, solid-phase extraction sorbent selection), chromatographic columns, and instrumental configurations, represents a major source of inconsistency among studies. Furthermore, these factors make it difficult to compare results across studies and regions. To address these issues, our study aimed to establish a standardized, practical workflow for rapid wastewater collection, extraction, and drug detection in wastewater, with a focus on minimizing analyte loss, reducing variability, and improving reproducibility. This study presents a validated and scalable method for close-to-real-time analysis of multiple drug residues in wastewater. Additional considerations are also discussed with a goal of providing a comprehensive set of factors to consider when establishing wastewater-based surveillance for drugs in a public health context.

## 2. Materials and Methods

### 2.1. Sample Collection

**Figure 1:**
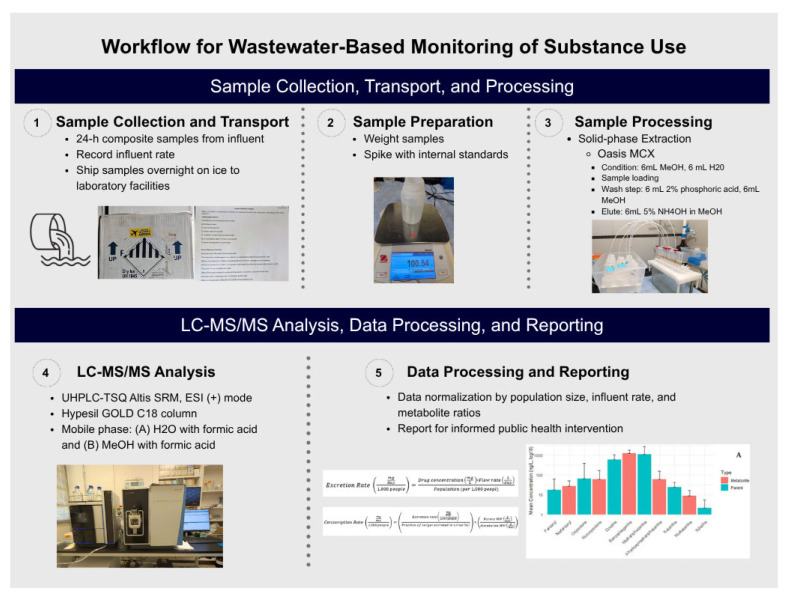
Overview of the standardized analytical workflow for wastewater-based monitoring of substance use, from sample collection through quantitative data re-porting.

Pooled influent wastewater samples from the University of Florida (UF) wastewater treatment facility (WWTF) were collected and used for method development and validation to ensure consistency across all experimental analyses. Wastewater samples were transported on wet ice or ice packs to the laboratory and divided into 100 mL aliquots, which were immediately used in spike recovery experiments. Additionally, 100 mL aliquots were used for quality assurance (see Section 2.3 for method validation details). An overview of the standardized analytical workflow from sample collection through quantitative data reporting is provided in Figure 1.

### 2.2. Sample Preparation

Each wastewater sample was gravimetrically weighed and spiked with 50 µL of an internal standard mixture containing 22 isotopically labeled compounds (listed in Table A1). Individual isotopically labeled internal standards (IS), purchased from Cayman Chemical, Ann Arbor, MI, USA, were first diluted to approximately 500 ng/mL in methanol. A secondary working stock was then prepared by further diluting this mixture to 50 ng/mL, which was used for sample spiking. In total, 52 native analytes, as shown in Table A1, were monitored in this method (Table A1), spanning major drug subclasses commonly detected in wastewater: opioids (*n* = 15), stimulants (*n* = 11), benzodiazepines (*n* = 5), synthetic cathinones (*n* = 5), dissociatives (*n* = 2), and other pharmaceuticals and metabolites (*n* = 14). These native standards were also purchased from Cayman Chemical, USA, and were spiked into both calibrants and quality control (QC) samples. Solvents and reagents used for extraction were Optima LC-MS grade methanol, ammonium hydroxide, water, and HPLC grade phosphoric acid (Fisher Scientific, Waltham, MA, USA). The wastewater samples were spiked with 100 µL of phosphoric acid. Once acidified, the samples were filtered via vacuum using 55 mm grade GF/A glass microfiber filters (Sigma Aldrich, St. Louis, MO, USA). Samples were then extracted via solid phase extraction (SPE) using Oasis MCX 60 µm mixed-mode polymeric cationic (Waters, Milford, MA, USA) exchange sorbent cartridges (500 mg/6 mL) from Waters. The cartridges were first conditioned with 6 mL of 5:95 ammonium hydroxide:methanol solution until saturation, soaked for 2 min, and passed through the cartridge by gravity. This process was repeated with 6 mL of Milli-Q water. This was followed by the loading and passing of the wastewater samples, dripping at a rate of 1–2 drops per second. The cartridges were then washed with 6 mL of 90:10 2% phosphoric acid to methanol buffer solution and dried under full vacuum for 50 min. Once the cartridges were dried, they were washed with 6 mL of methanol. Analytes were eluted using 6 mL of a 5% ammonium hydroxide solution in methanol and collected into 15 mL glass conical centrifuge tubes. Extracts were evaporated under ultra-high-purity nitrogen gas to a final volume of approximately 0.125 mL. Samples were then diluted with 0.375 mL of water to achieve a final volume of 0.5 mL. Reconstituted extracts were stored at −20 °C until analysis. For LC-MS/MS analysis, a 50 µL aliquot was transferred to a polypropylene autosampler vial for immediate injection.

In a preliminary evaluation, a subset of samples was also extracted using Oasis HLB cartridges (60 µm hydrophilic-lipophilic balance polymer sorbent; Waters, Milford, MA, USA) to compare recovery performance with the MCX extraction. These samples were not acidified prior to extraction. HLB cartridges were conditioned with 6 mL of 100% methanol followed by 6 mL of Milli-Q water. Sample loading and flow rate were consistent with the MCX protocol. Cartridges were then washed with 6 mL of 95:5 (*v*/*v*) water to methanol, dried under vacuum for 50 min, and eluted with 6 mL of 100% methanol. The eluates were evaporated and reconstituted in the same manner as the MCX samples. To test the selectivity of the cartridges, extraction efficiencies were determined by spiking half of the samples prior to extraction and the other half after extraction for both sorbents. Extraction efficiencies were calculated for each compound and for each of the sorbents by dividing the mean of the peak areas for three replicate extractions that were spiked before extraction by the mean of the peak areas of three replicate extractions that were spiked after extraction. The comparison of recovery and precision between MCX and HLB informed the selection of the MCX method for all subsequent sample processing, and cartridge performance differences are discussed in Section 3.

### 2.3. Analytical Method Development and Validation

The analysis of extracts was completed using a Thermo Scientific Vanquish Ultra-High Performance Liquid Chromatography (UHPLC) system, coupled to a TSQ Altis triple quadrupole mass spectrometer (Thermo Scientific, Waltham, MA, USA), with electrospray ionization in positive mode. The chromatographic separation was achieved using a Hypersil GOLD C18 column (150 mm × 2.1 mm, 5 µm; Thermo Scientific, Waltham, MA, USA). The mobile phases were composed of water (A) and methanol (B), both containing 0.1% of formic acid. The multistep chromatographic gradient employed was as follows: 0–0.5 min 1% B, 0.5–20 min 99% B, 20–23 99% B, 23–25 min 1% B, and 25–30 min 1% B. The temperature of the column compartment was set to 40 °C, with the autosampler temperature set to 4 °C. The flow rate was set to 0.2 mL/min, and the injection volume was 2 µL. Additional LC parameters are displayed in Table 1. The mass spectrometer was operated in positive selected reaction monitoring (SRM) mode using two transitions per species. The transition with the largest intensity was used for quantification, with the second transition used for confirmation. All precursor *m*/*z* ions for each species, along with their respective first and second transition *m*/*z* values and collision energies can be seen in Table A2. The positive mode source conditions were set to spray voltage 2000 V and sheath, auxiliary, and sweep gas being set to 50, 10, and 1 Arb, respectively. The temperatures of the ion transfer tube and vaporizer were set to 325 °C and 350 °C, respectively. Additional ion source parameters can be found in Table 1.

The calibrants were spiked with ranging native standard, 25 µL of the isotopically labeled internal standard mixture, and were diluted with 3:1 Optima Water and Optima methanol to achieve target concentrations. Quantitation was accomplished through isotope dilution using 22 calibration levels spanning from 0.001 ng/mL to 200 ng/mL. Calibration curves were constructed for each species, with each having an R^2^ > 0.995. For quality assurance, sample extracts, method blanks, wastewater as quality control (QC) samples, and calibrants were randomly distributed within the queue for analysis. The QCs samples consisted of a method blank (MilliQ water spiked with IS only) and two wastewater samples, one spiked with IS only, and one spiked with both IS and native stock. The resultant peak areas were obtained using Thermo Scientific TraceFinder software v 5.1. Limits of detection (LOD) and limits of quantitation (LOQ) for each native compound were determined visually for each extract based on signal-to-noise (S/N) ratios, with thresholds defined as 3:1 for LOD and 10:1 for LOQ. All samples were spiked with corresponding isotopically labeled internal standards prior to extraction to account for matrix effects and analytical variability during integration and quantification. The average of the first five LOD and LOQ determinations for each native compound was calculated and used as a reference value for subsequent analyses. For each run, if the LOD or LOQ was lower than the reference average, the average value was applied to maintain consistency. Conversely, if the LOD or LOQ exceeded the average, the higher value was used to adopt a more conservative approach. The average LOD and LOQ for each native compound are listed in Table A2. Final concentrations were normalized by the density of wastewater extracted and were reported in ng drug per L of wastewater (ng/L).

## 3. Results and Discussion

### 3.1. Sample Preparation

One of the most essential aspects of any analytical method is the extraction (e.g., preconcentration and purification of target compounds). In this study, the target analytes belong to different chemical classes, which results in varying physicochemical properties and a concomitant increase in the complexity of extraction. For example, some chemicals, such as cannabinoids, are neutral at lower pHs [18]. In our efforts to establish a standardized and practical workflow for multi-drug detection in wastewater, we compared two extraction methods to determine which one provided the best recovery for the majority of the target compounds. We optimized the solid-phase extraction (SPE) process by evaluating two distinct types of sorbents: the 6 mL Oasis HLB and the 6 mL Oasis MCX. The analytes extracted using the MCX cartridges showed adequate extraction efficiencies, ranging from 65% to 120%, with a few exceptions such as 2-Ethylidene-1,5-dimethyl-3,3-diphenylpyrrolidine, the primary metabolite of methadone, naproxen, propylhexedrine, phenacetin, and tianeptine. The mean extraction efficiency across all compounds using MCX was 82%, while HLB cartridges showed a higher mean efficiency of 103%. However, extraction variability differed substantially between the two sorbents: most HLB recoveries exhibited relative standard deviations (RSDs) greater than 20%, whereas MCX cartridges showed a markedly lower overall RSD of 15%. Although a few outliers reduced the overall mean efficiency for MCX, this sorbent provided more uniform recoveries across the analyte panel, which is critical for multi-residue methods. Given the lower variability and broader consistency observed with MCX, this sorbent was selected for further method development. Recoveries for all compounds obtained with both sorbents are presented in Table 2. Comparable findings have been reported by González-Mariño et al. (2009), who also noted improved performance and manageable signal suppression when using MCX, compared to HLB [19]. In their study, they reported recoveries of 451% and 112% for amphetamine and methamphetamine, respectively, using HLB and 118% and 101% using MCX. In our method, we report recoveries of 100% and 112% in HLB and 85% and 78% in MCX for the same drugs respectively.

### 3.2. Method Validation

Overall validation of the workflow was conducted prior to the application of the analytical procedure to the actual wastewater samples from the 17 sites explained in Section 3.3. This validation considered linearity, precision, accuracy, LODs, and LOQs. The following results are based on MCX cartridges as they were selected for usage in the extraction of the actual samples.

Calibration curves for all analytes had determination coefficients (R^2^) higher than 0.99. Additionally, recoveries (65–120%) and precision (calculated relative standard deviation (RSD) < 20%) were satisfactory for most compounds (>80%). Accuracy was calculated by dividing the measured concentration by the nominal concentration for each drug in the QC samples. All drugs spiked into the QCs were considered satisfactory when the measured concentrations were within ±20% of the expected values, and the relative standard deviation (RSD) below 20% [20]. The list of drugs, along with their accuracy, is presented in Table A3.

The LOD and LOQ for each compound were determined and visually confirmed. Several analytes, with high abundance in wastewater, typically had lower sensitivity (i.e., amphetamine and caffeine, with higher LODs and LOQs). Overall, the LOD and LOQ of all analytes were within the three lowest calibrants, which confirms the high sensitivity of the workflow to detect the compounds at very low ranges, down to 0.001 ng/mL LOD and 0.006 ng/mL LOQ. The set of calibration standards, ranging from 0.001 to 200 ng/mL, was run five times, and the average LOD and LOQ of those five calibration curves (shown in Table A2) were used as a reference for subsequent samples as detailed in Section 2.3.

### 3.3. Application to City Wastewater Samples

To demonstrate the performance and applicability of our validated method, we evaluated the occurrence of 52 drugs of abuse in influent wastewater collected from 17 sites across five major U.S. metropolitan areas using the multi-drug workflow described above. The selected analytes span multiple drug classes, enabling comprehensive assessment of community-level consumption patterns. These classes include stimulants, opioids, benzodiazepines and related sedatives, cocaine-related compounds, hallucinogens, other psychoactive substances (e.g., mitragynine), antivirals (e.g., oseltamivir), and non-psychoactive compounds (e.g., xylazine, 4-hydroxyxylazine, and pentachlorophenol).

**Figure 2:**
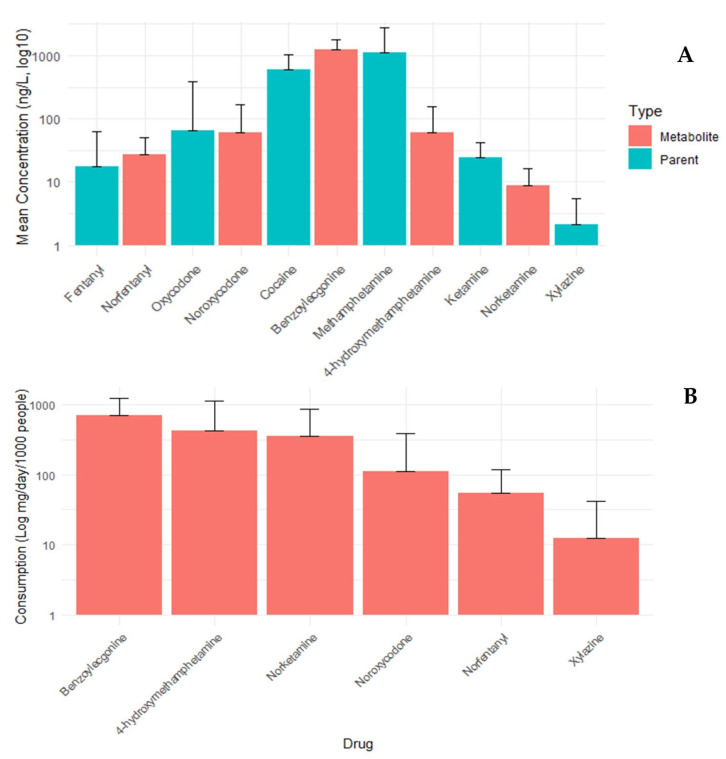
Average concentration (**A**) and consumption rate (**B**) of selected drugs (green) and their respective primary metabolite (orange) across all the 17 treatment facilities and weekly samples over the 9-month period for a total *n* of 598 for each drug.

Influent wastewater samples (250 mL, 24 h composites) were collected once per week over a 9-month period from the 17 participating wastewater treatment facilities (WWTFs) as part of the National Drug Early Warning System (NDEWS) project. Samples were shipped overnight to the laboratory on wet ice or ice packs immediately following collection. Concentrations of each drug (ng/L) were quantified for every facility and weekly sample (total *n* = 598) and subsequently used to calculate excretion and consumption rates using the equations in Appendix A.5, which were reported to the corresponding city authorities. To aid in interpretation, Figure 2A,B present the concentration and consumption rate, respectively, of several of the most frequently detected compounds from different drug classes, where each data point represents the mean across the entire dataset. Figure 2B depicts only metabolites, except for Xylazine given that the metabolite, 4-hydroxyxylazine, was inconsistently detected in the dataset. A complete list of targeted analytes and their concentration ranges in city wastewater samples is provided in Table A4.

### 3.4. Additional Considerations for Method Development

#### 3.4.1. Sample Handling and Preparation

Proper handling of wastewater samples is essential for ensuring the accuracy and reproducibility of analytical results, particularly when analyzing multiple drugs with varying chemical moieties and stability. Special attention should be given to sample collection and shipping, sample processing, and both short-term and long-term storage. During sample collection, it is recommended to use composite sampling over 24 h as this is more representative of community-level drug abuse and also minimizes temporal variability. Additionally, normalization techniques should be used to account for flow rates in catchment areas and population size. Once the samples have been collected, transportation must be performed rapidly and under cooled conditions to prevent microbial degradation and chemical transformation of the target analytes [10]. In our validated workflow, samples were shipped overnight on ice the same day as they were collected. Upon receipt of samples, they were immediately extracted or stored at −20 °C until further analysis.

Sample processing can become challenging when addressing multiple drugs, especially in complex matrices such as wastewater. While we aimed to provide a comprehensive approach, to encompass analysis of a wide myriad of drugs, for those drugs not detected well enough, methods tailored to their optimal conditions may need to be considered. For example, THC and its primary metabolite, THC-COOH, are highly hydrophobic and tend to adsorb to suspended solids in wastewater. As a result, sample filtration can reduce their recovery and detection in the dissolved phase [11,18], representing a limitation of the proposed standardized protocol when targeting cannabinoid biomarkers. Additionally, raw samples should be stored in an environment at or below −20 °C, but ideally at −80 °C, especially for long-term storage. For example, a study on the effect of temperature storage in wastewater degradation showed little variation in water physicochemical parameters among 4, −20, and 80 °C up to 12 weeks; however, these parameters varied significantly among temperatures after 12 weeks [21]. Continuous freeze–thaw cycles could also compromise sample integrity; thus, those cycles should be minimized as much as possible [21]. Overall, multiple considerations need to be evaluated throughout the entire process of analyzing wastewater samples for illicit drug monitoring and surveillance.

When developing methods to analyze a broad range of drugs in complex matrices like wastewater, several key factors must be considered. These include selecting an appropriate extraction approach to effectively concentrate the analytes of interest. This is done by selecting the appropriate type of SPE cartridge based on analyte characteristics, or solvent system if using liquid–liquid extraction (LLE). Each extraction technique has tradeoffs; for example, LLE may provide broader selectivity of compounds, but may also result in decreased sensitivity due to ion suppression from matrix. Additionally, the optimization of the mobile phase composition for LC-MS analysis must be considered when developing a multi-drug method. While our study focused on solid-phase extraction (SPE), other studies have successfully employed LLE methods [19,22,23]. Most studies utilize an SPE method, though some studies have explored the use of different cartridges such as HLB or weak anionic exchange (WAX) sorbents [24]. Though successful in their extraction selectivity, these previous studies targeted fewer drugs in their method and focused more on select drug classes. Additionally, with the use of these different cartridges, different elution solvents such as NH_3_/MeOH, MeOH/ethyl acetate, chloroform, and acetonitrile/acetic acid were used [25,26].

#### 3.4.2. Mobile Phase and Column Selection

In our study, a gradient of methanol and water (both with 0.1% formic acid) was utilized with success; however, other studies have also used different mobile phases, such as acetonitrile or acetonitrile with acetic acid [26,27]. Selection of appropriate mobile phase and gradient conditions will depend on column selection, required chromatographic resolution, and compound compatibility.

The choice of analytical column plays a critical role in the overall performance of LC-MS/MS methods for drug detection in wastewater. The Hypersil GOLD C18 column (150 mm × 2.1 mm, 5 µm; Thermo Scientific, Waltham, MA, USA) was selected for its robust performance in separating a broad spectrum of analytes with varying polarity and structural complexity [28], which makes it an ideal candidate for our multi-drug approach. The C18 stationary phase is widely used in drug analysis due to its compatibility with both polar and non-polar compounds and its high reproducibility. Moreover, C18 provided sufficient resolution and peak shape across the drug classes detected, though alternative columns such as phenyl-hexyl, biphenyl, or polar-embedded phases are sometimes used to enhance retention or resolution of specific drug classes (e.g., synthetic cannabinoids, stimulants, or highly polar metabolites) [29]. Our method demonstrated adequate chromatographic separation, with analyte retention times ranging from 7 to 19 min (Table A2). Data were acquired using a TSQ Altis Plus triple quadrupole mass spectrometer (Thermo Scientific, Waltham, MA, USA) operated in multiple reaction monitoring (MRM) mode, with acquisition speeds of up to approximately 600 transitions per second. For multi-drug methods, further improvements in resolution may be achieved by optimizing acquisition parameters, including scan speed and the number of monitored transitions.

#### 3.4.3. Reporting Metrics

When utilizing wastewater surveillance for drug use, compound concentration in the sample may not allow for comparison across sites if flow rates across sites are considerably different. In these cases, normalized excretion rates for each compound can be calculated and reported by adjusting for the population served by each WWTF catchment area and the corresponding wastewater flow rate at the time of sample collection [30]. Another key reporting metric is the consumption rate of each compound, in which these rates are estimated by adjusting calculated excretion values using pharmacokinetic correction factors. Specifically, the excretion rate is divided by the fraction of the compound excreted in urine. When metabolites are measured, instead of the parent compounds, the excretion rate is further adjusted by multiplying it by the ratio of the parent compound’s molecular weight to that of the metabolite, to account for biotransformation. The equations used to back-calculate both excretion and consumption rates are provided in Appendix A.5. It is important to note several uncertainties and assumptions involved in estimating these rates. For instance, weather patterns can affect flow rates and/or more touristic areas may be influenced by visitors during certain months of the year, affecting the accuracy of per capita consumption estimates [30]. Nevertheless, the presence of certain compounds in wastewater may not necessarily imply local consumption, as contributions may also come from non-residential sources such as commuters, hospitals, or industrial facilities, potentially affecting the representativeness of the data.

## 4. Conclusions

This study reaffirms the utility of wastewater-based epidemiology as a cost-effective and non-invasive approach for monitoring community drug use trends, with the potential to inform public health interventions and guide policy development. The methodology demonstrated here is scalable and sustainable, offering strong potential for long-term surveillance and timely public health response to emerging drug trends.

This work contributes to the development of a standardized protocol for multi-drug detection in wastewater, addressing critical methodological components from sample collection to analytical data interpretation. A key finding was the importance of sorbent selection during sample processing, with Oasis MCX cartridges being selected for their robust performance across a broad range of compounds with diverse physicochemical properties, providing consistently high recovery, accuracy, and precision compared to Oasis HLB. Furthermore, this framework is designed to be adaptable to specific drug classes. For instance, cannabinoid recovery may be improved by omitting filtration prior to SPE to minimize analyte loss, as well as by evaluating alternative analytical columns with greater compound affinity.

Additional considerations include proper sample collection strategies (e.g., 24 h composite sampling), rapid and temperature-controlled transport, careful processing, optimal storage conditions, and important reporting metrics such as excretion and consumption rates. Together, this research supports the use of LC-MS/MS and WBE as a valuable tool in public health surveillance and policy development. Moreover, WBE combined with hospitalizations and clinical survey data could enhance the understanding of opioid and substance use, improving public health interventions. Lastly, in addition to the inter-regional validation presented in the current framework, international collaborations, such as those described in [31], represent important ongoing efforts to harmonize methodologies, expand validation across diverse settings, and further enhance the applicability and impact of WBE in public health monitoring.

## Figures and Tables

**Table 1:** LC and ion source parameters.

LC Conditions (Thermo Scientific Vanquish UHPLC)	
Column	Hypersil GOLD Thermo Scientific C18 150 mm × 2.1 mm; 5 µm
Mobile phases	Water (A) and methanol (B), both containing 0.1% formic acid
Gradient elution	0–20 min 1% B, 20–23 min 99% B, 23–25 min 1% B, and 25–30 min 1% B
Flow rate	0.2 mL/min
Column temperature	40 °C
Autosampler temperature	4 °C
Injection volume	2 µL
**Ion Source Parameters (Thermo Altis MS/MS)**	
Ion source type	H-ESI
Spray voltage	Static
Positive ion	2000 V
Sheath gas	50 Arb
Aux gas	10 Arb
Sweep gas	1 Arb
Ion transfer tube temperature	325 °C
Vaporizer temp	350 °C

**Table 2:** Comparison of analyte recoveries from solid phase extraction using Oasis HLB and Oasis MCX cartridges.

Drug	HLB in Wastewater		MCX in Wastewater	
	Recovery (%)	RSD (%)	Recovery (%)	RSD (%)
3,4-Methylenedioxymethamphetamine	112	23	62	8
3,4-Methylenedioxypyrovalerone	106	22	94	12
4-hydroxymethamphetamine	122	23	87	11
4-hydroxyxylazine	101	29	84	13
4-hydroxyxylazine-d_6_	96	27	85	13
6-acetylmorphine	130	22	109	8
Alpha-Pyrrolidinopentiophenone	103	22	83	11
Alprazolam	111	24	71	11
Amphetamine	100	19	85	10
Amphetamine-d_8_	78	18	74	9
Benzoylecgonine	125	20	90	12
Benzoylecgonine-d_3_	221	16	82	11
Buprenorphine	94	22	87	12
Buprenorphine-d_9_	86	20	87	13
Caffiene	115	19	99	11
Carfentanil	103	25	100	12
Clonazepam	113	25	82	12
Cocaethylene	103	23	91	11
Cocaine	97	22	95	11
Cocaine-d_3_	90	21	94	12
Codeine	107	26	95	11
Desomorphine	112	23	95	11
EDDP	102	21	19	65
EDDP-d_3_	92	19	15	65
Ethylone/Bk-MDEA	111	24	86	11
Etizolam	112	22	85	12
Etonitazene	82	22	86	10
Etonitazene-13C_6_	78	22	86	11
Eutylone	109	22	91	10
Fentanyl	104	21	89	10
Fentanyl-d_5_	100	20	88	11
Flualprazolam	115	22	70	10
Flunitrazepam	107	23	76	11
Gabapentin	51	43	96	9
Heroin	82	25	N/A	N/A
Heroin-d_3_	77	22	N/A	N/A
Isotonitazene	72	25	85	11
Isotonitazene-13C_6_	68	22	86	12
Ketamine	113	23	90	11
Ketamine-d_4_	102	22	91	12
Methadone	105	23	89	11
Methadone-d_9_	98	21	89	12
Methamphetamine	112	22	78	9
Methamphetamine-d_8_	103	20	72	8
Methaqualone	112	22	89	10
Methylphenidate	97	22	94	10
Mitragynine	80	23	84	21
N,N-Dimethylpentylone	106	22	91	10
Naloxone	190	25	84	13
Naloxone-d_5_	175	23	83	12
Norbuprenorphine	100	18	91	10
Norbuprenorphine-d_3_	92	16	93	12
Norfentanyl	97	22	94	12
Norfentanyl-d_5_	90	17	99	12
Norketamine	105	21	92	12
Norketamine-d_4_	97	21	92	12
Noroxycodone	113	21	92	12
Noroxycodone-d_3_	97	21	79	12
Oseltimivir	91	17	82	13
Oxycodone	121	24	90	11
Oxycodone-d_3_	111	24	87	12
para-Fluorofentanyl	101	21	90	11
Phenylcyclohexyl piperidine	111	23	85	11
Propylehexedrine	109	21	34	8
Xylazine	98	25	88	12
Xylazine-d_6_	90	24	86	12
Zolpidem	111	23	93	10
Average Across All Species	103	22	82	14

## Data Availability

The datasets presented in this article are not readily available because they contain location-specific information and are part of an effort to maintain the anonymity of the cities included in the study. Requests to access the datasets should be directed to the corresponding author.

## Abbreviations

The following abbreviations are used in this manuscript:

SPE	Solid Phase Extraction
LLE	Liquid–Liquid Extraction
IS	Internal Standard
LOD	Limit of Detection
LOQ	Limit of Quantification
SRM	Selected Reaction Monitoring
MRM	Multiple Reaction Monitoring
QC	Quality Control
UHPLC	Ultra-High Performance Liquid Chromatography
MCX	Mixed-mode Cation eXchange
HLB	Hydrophilic-Lipophilic Balanced
WBE	Wastewater-Based-Epidemiology
WWTF	WasteWater Treatment Facility
LC-MS/MS	Liquid Chromatography-Tandem Mass Spectrometry

## Appendix A

### Appendix A.1

**Table A1:** List of drugs analyzed along with their corresponding isotope-labeled internal standards.

Drug	Formula	Corresponding Isotopically Labeled Standard
3,4-Methylenedioxymethamphetamine (MDMA)	C_11_H_15_NO_2_	Methamphetamine-d_8_
3,4-Methylenedioxypyrovalerone (MDPV)	C_16_H_21_NO_3_	Cocaine-d_3_
4-hydroxymethamphetamine	C_10_H_15_NO	4-hydroxymethphetamine-d_3_
4-hydroxyxylazine	C_12_H_16_N_2_OS	4-hydroxyxylazine-d_6_
6-acetylmorphine	C_19_H_21_NO_4_	Amphetamine-d_8_
Alpha-pyrrolidinopentiophenone (Alpha-PPV)	C_15_H_21_NO	Cocaine-d_3_
Alprazolam	C_17_H_13_ClN_4_	Methadone-d_9_
Amphetamine	C_9_H_13_N	Amphetamine-d_8_
Benzoylecgonine	C_16_H_19_NO_4_	Benzoylecgonine-d_3_
Buprenorphine	C_29_H_41_NO_4_	Buprenorphine-d_9_
Caffeine	C_8_H_10_N_4_O_2_	Methamphetamine-d_8_
Carfentanil	C_24_H_30_N_2_O_3_	Fentanyl-d_5_
Clonazepam	C_15_H_10_ClN_3_O_3_	Methadone-d_9_
Cocaethylene	C_18_H_23_NO_4_	Fentanyl-d_5_
Cocaine	C_17_H_21_NO_4_	Cocaine-d_3_
Codeine	C_18_H_21_NO_3_	4-hydroxyxylazine-d_6_
Desomorphine	C_17_H_21_NO_2_	Amphetamine-d_8_
2-Ethylidene-1,5-dimethyl-3,3-diphenylpyrrolidine (EDDP)	C_20_H_23_N	EDDP-d_3_
Ethylone/Bk-MDEA	C_12_H_15_NO_3_	Amphetamine-d_8_
Etizolam	C_17_H_15_ClN_4_S	Methadone-d_9_
Etonitazene	C_22_H_28_N_4_O_3_	Etonitazene-13C_6_
Eutylone	C_13_H_17_NO_3_	Methamphetamine-d_8_
Fentanyl	C_22_H_28_N_2_O	Fentanyl-d_5_
Flualprazolam	C_17_H_12_ClFN_4_	Methadone-d_9_
Flunitrazepam	C_16_H_12_FN_3_O_3_	Methadone-d_9_
Gabapentin	C_9_H_17_NO_2_	Amphetamine-d_8_
Heroin	C_21_H_23_NO_5_	Heroin-d_3_
Isotonitazene	C_23_H_30_N_4_O_3_	Isotonitazene-13C_6_
Ketamine	C_13_H_16_ClNO	Ketamine-d_4_
Methadone	C_21_H_27_NO	Methadone-d_9_
Methamphetamine	C_10_H_15_N	Methamphetamine-d_8_
Methaqualone	C_16_H_14_N_2_O	Methadone-d_9_
Methylphenidate	C_14_H_19_NO_2_	Cocaine-d_3_
Mitragynine	C_23_H_30_N_2_O_4_	Fentanyl-d_5_
N,N-Dimethylpentylone	C_14_H_19_NO_3_	Cocaine-d_3_
Naloxone	C_19_H_21_NO_4_	Naloxone-d_5_
Norbuprenorphine	C_25_H_35_NO_4_	Norbuprenorphine-d_3_
Norfentanyl	C_14_H_20_N_2_O	Norfentanyl-d_5_
Norketamine	C_12_H_14_ClNO	Norketamine-d_4_
Noroxycodone	C_17_H_19_NO_4_	Noroxycodone-d_3_
Oseltimivir	C_16_H_28_N_2_O_4_	Methadone-d_9_
Oxycodone	C_18_H_21_NO_4_	Oxycodone-d_3_
para-Fluorofentanyl	C_22_H_27_FN_2_O	Fentanyl-d_5_
Phenylcyclohexyl Piperidine (PCP)	C_17_H_25_N	Fentanyl-d_5_
Phenacetin	C_10_H_13_NO_2_	Fentanyl-d_5_
Propylehexedrine	C_10_H_21_N	Fentanyl-d_5_
Xylazine	C_12_H_16_N_2_S	Xylazine-d_6_
Zolpidem	C_19_H_21_N_3_O	Cocaine-d_3_

### Appendix A.2

**Table A2:** List of drugs with corresponding instrument parameters, including retention time, precursor and product ions, collision energy, RF lens voltage, and limits of detection and quantification.

Drug	Retention Time (min)	Precursor (m/z)	Product (m/z)	Collision Energy (V)	RF Lens (V)	Mean LOD	Mean LOQ
3,4-Methylenedioxymethamphetamine	10.46	193.963	104.967	25.27	31	0.001	0.003
3,4-Methylenedioxypyrovalerone	12.93	276.05	125.967	26.98	60	0.004	0.007
4-hydroxymethamphetamine	7.26	165.963	106.883	21.68	30	0.021	0.021
4-hydroxymethamphetamine-d_3_	7.26	169.05	106.883	21.76	30	N/A ^1^	N/A ^1^
4-hydroxyxylazine	9.66	237.047	89.883	24.19	69	0.005	0.007
4-hydroxyxylazine-d_6_	9.66	243.05	89.883	25.27	70	N/A ^1^	N/A ^1^
6-acetylmorphine	10.03	328.13	164.967	41.16	77	0.006	0.021
Alpha-Pyrrolidinopentiophenone	12.72	232.047	90.883	25.55	55	0.004	0.019
Alprazolam	18.5	308.987	204.883	43.31	89	0.02	0.02
Amphetamine	10.02	136.01	90.967	18.61	30	0.236	0.329
Amphetamine-D_8_	10.02	144.06	96.967	17.82	30	N/A ^1^	N/A ^1^
Benzoylecgonine	12.4	290.05	76.883	50.33	57	0.006	0.041
Benzoylecgonine-d_3_	12.4	293.047	76.883	50.61	60	N/A ^1^	N/A ^1^
Buprenorphine	15.22	468.273	54.967	47.18	104	0.003	0.021
Buprenorphine-D_9_	15.22	477.333	54.967	46.96	104	N/A ^1^	N/A ^1^
Caffiene	11.47	194.917	137.883	19.61	59	0.25	0.25
Carfentanil	16.19	395.13	335.133	17.96	67	0.007	0.022
Clonazepam	17.75	316	213.883	39.16	83	0.006	0.019
Cocaethylene	13.72	318.1	81.883	32.07	60	0.003	0.006
Cocaine	12.56	304.047	104.883	33.07	60	0.001	0.006
Cocaine-d_3_	12.56	307.05	185.05	19.89	59	N/A ^1^	N/A ^1^
Codeine	9.18	299.85	164.967	45.17	57	0.006	0.025
Desomorphine	10.01	272.117	166.967	39.23	70	0.055	0.067
EDDP	15.07	278.13	233.967	31.42	74	0.001	0.003
EDDP-D_3_	15.07	281.18	233.967	31.85	75	N/A ^1^	N/A ^1^
Ethylone/Bk-MDEA	10.19	222.05	173.967	18.82	48	0.005	0.018
Etizolam	18.76	342.967	258.883	36.15	82	0.003	0.005
Etonitazene	15.42	397.24	71.967	36.44	75	0.001	0.003
Etonitazene-13C_6_	15.42	403.2	71.967	36.87	74	N/A ^1^	N/A ^1^
Eutylone	11.41	236.047	187.967	18.46	45	0.019	0.039
Fentanyl	15.38	337.187	104.967	38.44	67	0.001	0.005
Fentanyl-d_5_	15.38	343.213	104.967	39.37	68	N/A ^1^	N/A ^1^
Flualprazolam	18.11	327.1	298.883	27.84	82	0.006	0.043
Flunitrazepam	17.82	314.047	238.967	35.79	80	0.005	0.007
Gabapentin	10.06	172	136.967	16.6	31	0.023	0.046
Heroin	12.39	370.053	164.883	52.12	85	N/A ^1^	N/A ^1^
Heroin-d_3_	12.39	373.133	164.883	52.33	85	N/A ^1^	N/A ^1^
Isotonitazene	16.21	411.27	71.967	37.8	75	0.001	0.003
Isotonitazene-13C_6_	16.21	417.23	71.967	38.37	75	N/A ^1^	N/A ^1^
Ketamine	11.75	238.025	124.883	28.77	40	0.001	0.03
Ketamine-D_4_	11.75	242.05	128.883	29.56	41	N/A ^1^	N/A ^1^
Methadone	16.72	310.167	104.967	28.77	47	0.003	0.003
Methadone-d_9_	16.72	319.217	104.883	29.28	49	N/A ^1^	N/A ^1^
Methamphetamine	10.35	150.03	90.883	20.83	30	0.023	0.049
Methamphetamine-D_8_	10.35	158.047	92.967	21.11	30	N/A ^1^	N/A ^1^
Methaqualone	17.87	251.047	90.967	42.17	74	0.001	0.004
Methylphenidate	12.67	234.05	55.883	43.24	46	0.003	0.005
Mitragynine	14.67	399.133	173.967	32.14	80	0.003	0.006
N,N-Dimethylpentylone	5.29	286.057	151.883	63	71	0.003	0.005
Naloxone	9.1	328.133	211.917	40.733	61	0.005	0.022
Naloxone-d_5_	9.1	333.133	211.883	41.59	63	N/A ^1^	N/A ^1^
Norbuprenorphine	14.13	414.193	186.967	39.66	87	0.006	0.052
Norbuprenorphine-D_3_	14.13	417.203	186.967	40.37	90	N/A ^1^	N/A ^1^
Norfentanyl	12.12	233.05	54.883	35.86	42	0.003	0.005
Norfentanyl-d_5_	12.12	238.047	54.967	36.51	42	N/A ^1^	N/A ^1^
Norketamine	11.71	224.04	124.883	26.55	30	0.009	0.03
Norketamine-D_4_	11.71	228.02	128.883	26.41	30	N/A ^1^	N/A ^1^
Noroxycodone	9.73	302.05	186.967	25.7	52	0.042	0.247
Noroxycodone-d_3_	9.73	305.1	189.967	26.27	51	N/A ^1^	N/A ^1^
Oseltimivir	15.4	313.05	165.967	19.11	45	0.006	0.042
Oxycodone	9.54	316.05	240.967	29.63	60	0.022	0.045
Oxycodone-d_3_	9.54	319.133	243.967	30.35	60	N/A ^1^	N/A ^1^
para-Fluorofentanyl	15.59	355.157	188.05	24.33	72	0.003	0.019
Phenylcyclohexyl piperidine	14.17	244.05	85.967	13.09	30	N/A ^1^	N/A ^1^
Propylehexedrine	13.92	156.047	54.967	28.27	30	0.004	0.019
Xylazine	12.03	221.057	89.883	23.26	63	0.001	0.005
Xylazine-d_6_	12.03	227.047	169.967	27.7	66	N/A ^1^	N/A ^1^
Zolpidem	13.06	308.13	262.967	26.41	80	0.002	0.004

^1^ No limit of detection and limit of quantification were calculated for internal standards.

### Appendix A.3

**Table A3:** Accuracy and standard deviation for each drug calculated based on native and internal standard quality controls.

Drug	Accuracy (%)	SD (%)
3_4-MDMA	84	1
3_4-MDPV	98	6
4-hydroxymethamphetamine	97	3
4-hydroxyxylazine	105	4
6-acetylmorphine	105	2
Alpha-PPV	103	8
Alprazolam	73	3
Amphetamine	167	85
Benzoylecgonine	*	*
Buprenorphine	75	4
Caffiene	*	*
Carfentanil	161	9
Clonazepam	54	0.25
Cocaethylene	127	10
Cocaine	86	33
Codeine	115	2
Desomorphine	61	2
EDDP	137	25
Ethylone/Bk-MDEA	81	2
Etizolam	71	3
Etonitazene	99	3
Eutylone	103	6
Fentanyl	121	6
Flualprazolam	72	2
Flunitrazepam	50	1
Gabapentin	*	*
Heroin	103	16
Isotonitazene	96	4
Ketamine	99	9
Methadone	102	6
Methamphetamine	119	22
Methaqualone	53	2
Methylphenidate	120	6
Mitragynine	83	5
N,N-Dimethylpentylone	104	6
Naloxone	107	9
Norbuprenorphine	81	9
Norfentanyl	102	4
Norketamine	107	5
Noroxycodone	110	41
Oseltimivir	66	7
Oxycodone	72	12
para-Fluorofentanyl	138	10
Phenylcyclohexylpiperidine	116	10
Propylehexedrine	57	6
Xylazine	96	5
Zolpidem	136	6

* Accuracy could not be calculated due to high background concentrations.

### Appendix A.4

**Table A4:** Summary statistics of drug concentrations measured in actual city samples.

Drug	n	Mean	Median	Min	Max	SD
3_4-MDMA	227	30.21	18.99	0.86	557.99	46.98
3_4-MDPV	10	0.18	0.18	0.06	0.26	0.06
4-hydroxymethamphetamine	598	62.40	21.17	0.71	510.99	100.11
4-hydroxyxylazine	389	0.16	0.08	0.02	2.15	0.22
6-acetylmorphine	326	1.53	1.09	0.05	13.63	1.54
Alpha-PVP	6	0.18	0.17	0.12	0.28	0.07
Alprazolam	186	0.83	0.70	0.13	4.03	0.61
Amphetamine	593	243.43	193.21	9.42	4787.22	349.19
Benzoylecgonine	598	1258.71	1216.71	87.30	3602.03	585.93
Buprenorphine	587	16.73	7.31	0.74	1385.76	61.25
Caffiene	227	13,297.63	10,730.50	1528.22	53,951.10	8565.68
Carfentanil	1	0.19	0.19	0.19	0.19	N/A ^1^
Clonazepam	1	0.18	0.18	0.18	0.18	N/A ^1^
Cocaethylene	227	13.13	12.01	0.98	41.09	7.48
Cocaine	598	625.81	593.51	21.23	3099.92	419.39
Codeine	596	180.23	37.33	3.32	34,073.55	1632.97
Desomorphine	33	1.16	0.99	0.17	3.12	0.76
EDDP	598	95.40	72.11	9.20	1655.38	88.01
Ethylone/Bk-MDEA	1	0.20	0.20	0.20	0.20	N/A ^1^
Etizolam	16	0.12	0.05	0.01	0.80	0.19
Etonitazene	56	0.09	0.07	0.01	0.34	0.07
Eutylone	145	0.56	0.41	0.09	5.61	0.59
Fentanyl	597	17.63	8.52	0.54	813.22	45.50
Flualprazolam	11	0.25	0.26	0.11	0.43	0.11
Gabapentin	227	16,417.61	13,528.12	2310.98	53,849.18	9315.51
Isotonitazene	102	0.06	0.03	0.01	0.42	0.08
Ketamine	598	24.73	20.77	0.80	154.78	17.91
Methadone	597	90.45	23.68	2.35	13,987.57	630.09
Methamphetamine	598	1102.51	469.87	31.35	8662.50	1692.84
Methaqualone	48	0.02	0.01	0.01	0.08	0.02
Methylphenidate	227	56.90	4.92	0.29	10,968.79	727.83
Mitragynine	227	17.94	9.82	1.86	218.83	21.45
N,N-Dimethylpentylone	125	5.37	0.14	0.01	78.10	14.95
Naloxone	417	20.73	3.79	0.20	1782.36	129.29
Norbuprenorphine	491	15.48	7.49	0.52	1495.93	73.78
Norfentanyl	598	27.22	21.43	1.45	143.48	22.95
Norketamine	592	9.02	6.57	0.33	82.82	8.05
Noroxycodone	592	56.56	39.27	2.49	1151.58	91.44
Oseltimivir	192	3.45	1.77	0.10	26.25	4.14
Oxycodone	592	53.98	21.42	1.34	4037.11	263.12
para-Fluorofentanyl	590	5.53	3.21	0.23	422.27	18.68
Phenylcyclohexyl piperidine	118	1.58	1.13	0.13	6.20	1.37
Propylehexedrine	137	0.34	0.29	0.05	1.14	0.24
Xylazine	598	2.06	0.94	0.08	28.06	3.40
Zolpidem	206	0.61	0.51	0.05	4.64	0.48

^1^ Standard deviation (SD) was not able to be calculated when the drug was detected only once.

### Appendix A.5

Excretion rates are calculated according to the following equation:

(A1) $$Excretion\;Rate\left(\frac{\frac{mg}{day}}{1000\;people}\right)=\frac{Drug\;concentration\left(\frac{mg}{L}\right)\times low\;rate\left(\frac{L}{day}\right)}{Population\;(per\;1000\;people)}$$

Consumption rates are calculated according to the following equation:

(A2) $$Consumption\;Rate\;\left(\frac{\frac{mg}{day}}{1000\;people}\right)=\left(\frac{Excretion\;rate\;\left(\frac{\frac{mg}{day}}{1000\;people}\right)}{Fraction\;of\;target\;excreted\;in\;urine\;\left(\%\right)}\right)\times \left(\frac{Parent\;MW\;\left(\frac{g}{mol}\right)}{Metabolite\;MW\;\left(\frac{g}{mol}\right)}\right)$$
